# Long-Term Weight Loss and Attendance Outcomes Following Metabolic and Bariatric Surgery: An Evaluation of The Cleveland Clinic Behavioral Rating System

**DOI:** 10.1007/s11695-024-07425-9

**Published:** 2024-10-10

**Authors:** Anne Jacobs, Karlijn Vermeer, Anna N. Slok, Ignace M. C. Janssen, Rob A. E. M. Tollenaar, Valerie M. Monpellier

**Affiliations:** 1 Nederlandse Obesitas Kliniek (Dutch Obesity Clinic), Amersfoortseweg 43, 3712 BA Huis Ter Heide, The Netherlands; 2 Department of Surgery, Leiden University Medical Center, Albinusdreef 2, 2333 ZA Leiden, The Netherlands

**Keywords:** Bariatric surgery, Metabolic surgery, Weight loss, Psychological factors, Cleveland Clinic

## Abstract

**Introduction:**

Interdisciplinary guidelines recommend preoperative psychological evaluation before metabolic and bariatric surgery (MBS). The Cleveland Clinic Behavioral Rating System (CCBRS) has been developed to evaluate the psychological state of individuals undergoing MBS. However, its predictive value concerning long-term weight loss and follow-up attendance has not been extensively studied. This study aims to assess the predictive value of the CCBRS regarding weight loss and follow-up attendance up to 5 years after MBS.

**Methods:**

In this cohort study (*n* = 1236), psychologists administered the CCBRS to each patient prior to MBS in addition to the standard psychosocial-behavioral screening. The CCBRS consists of nine psychological domains and is scored on a five-point Likert scale, from “poor” to “excellent.” Linear mixed models and ordinal regression analysis were used to analyze the percentage total weight loss over time and follow-up attendance up to 5 years after surgery.

**Results:**

A total of 1086 patients underwent subsequent MBS. Significant differences in weight loss and follow-up attendance were observed between some CCBRS groups compared to the reference group “excellent.” However, these differences were not consistent across all groups within any given domain.

**Conclusion:**

In this cohort, the predictive value of the CCBRS for weight loss and follow-up attendance up to 5 years after MBS was limited. It is important to consider certain limitations, such as considerable loss to follow-up. Nevertheless, the CCBRS remains valuable for structured psychological assessments by helping to identify patients’ strengths and areas needing improvement.

**Graphical Abstract:**

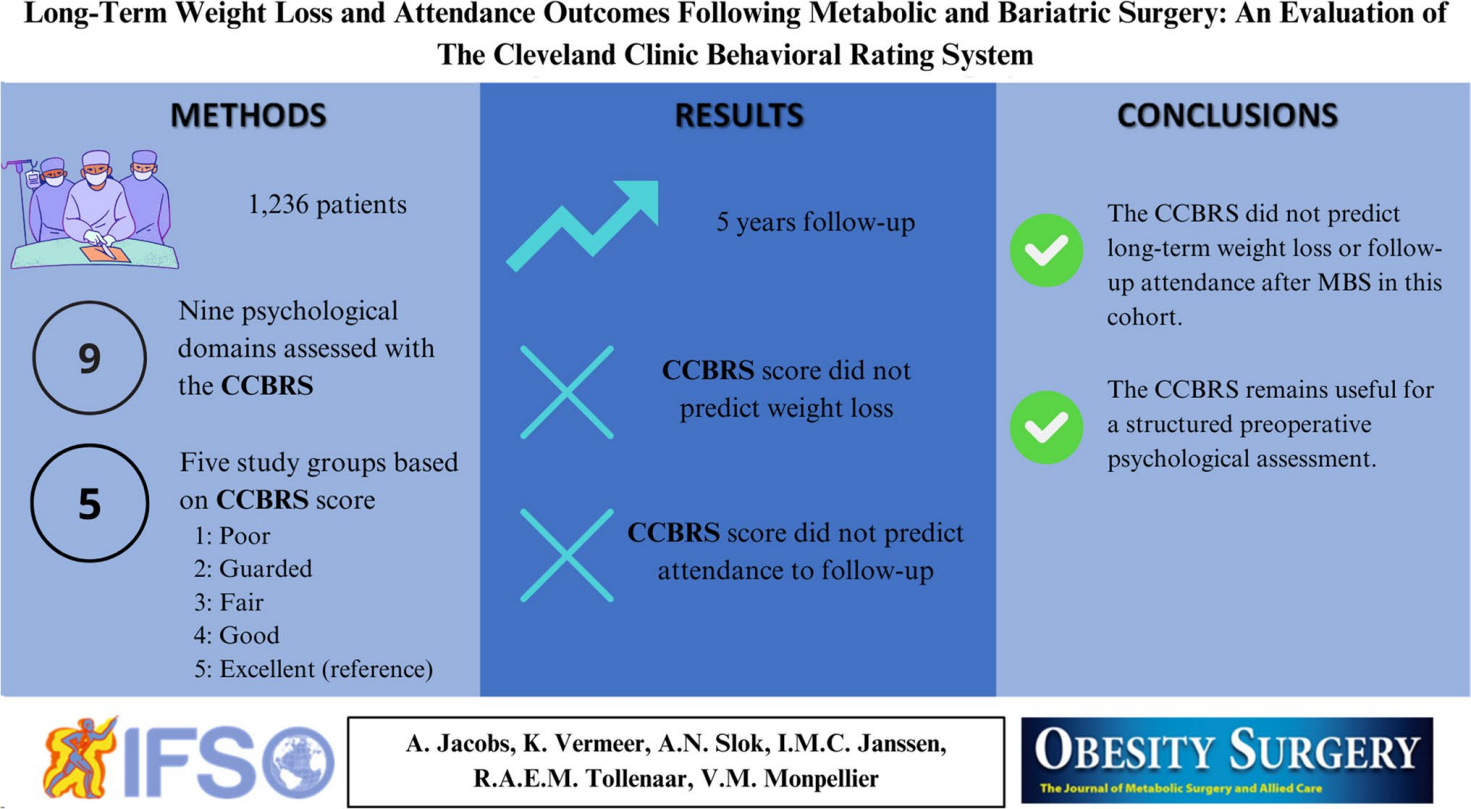

## Introduction

International guidelines on metabolic and bariatric surgery (MBS) recommend that all patients who are referred for MBS undergo a psychological evaluation prior to the procedure to identify any potential vulnerabilities and areas of concern that may impact treatment outcomes and long-term adherence, as well as to enable appropriate postoperative monitoring [1, 2]. The guidelines also outline what elements should be included in this psychological assessment. However, the specific implementation of these guidelines varies between institutions, and a variety of tools are available for assessing multiple domains of concern [3].

The Cleveland Clinic has developed a comprehensive instrument for evaluating the psychological state of individuals seeking MBS, known as the Cleveland Clinic Behavioral Rating System (CCBRS) [4]. The goal of the CCBRS is to provide a succinct summary of the patient’s strengths and areas for improvement, with a particular emphasis on assessing their “readiness” for the procedure, instead of delivering a final decision on whether they meet the necessary requirements. This system consists of nine discrete domains, including eight psychosocial domains (consent, expectations, social support, adherence, coping/stressors, mental health, substance use/abuse/dependence, eating behaviors), and an overall impression domain.

Although the CCBRS was not originally designed for the prediction of postoperative weight loss or attendance to follow-up, it has been investigated in prior studies, showing varying outcomes [4–6]. The CCBRS was found to be a useful instrument for the psychosocial assessment among 389 patients undergoing MBS (Roux-en-Y gastric bypass (RYGB), sleeve gastrectomy, and laparoscopic adjustable gastric banding) in the United States [4]. In this study, the CCBRS predicted the duration of hospital stay, but did not predict postoperative weight loss up to 1 year after MBS. Another study in the United States included 179 patients and illustrated that the domains “social support,” “coping,” and “overall impression” were associated with better attendance to follow-up, while only the domain “social support” had a significant association with weight loss 2 years after MBS [5]. In contrast, a recent study by the same author solely focused on the CCBRS domain “social support” and found no association with postoperative weight loss or attendance at 2-year follow-up [6]. Researchers advised further research should assess the predictive utility of the tool for longer term postoperative weight loss [4]. The CCBRS was implemented in the Dutch guideline for Bariatric psychology to facilitate structured psychodiagnostic screening aimed at identifying risk factors that may impede optimal weight loss outcomes or may elevate the likelihood of postoperative psychological complications [7].

Thus, the CCBRS is believed to be a useful tool for the preoperative psychological evaluation of individuals undergoing MBS. However, no previous research evaluated the predictive value of the CCBRS on longer term weight loss and attendance to follow-up after MBS in a large study population. Therefore, the aim of this study is to evaluate the predictive value of the CCBRS on weight loss and attendance up to 5 years after MBS in a Dutch sample.

## Methods

### Patient and Data Selection

In this prospective cohort study, all participants were screened between February 2016 and July 2017 at one location of a multicenter outpatient clinic for MBS in The Netherlands. The treatment at the clinic comprises a comprehensive pre- and postoperative counseling program [8]. The preoperative program consists of six group sessions conducted over a 6-week period prior to surgery, and the postoperative program comprises eleven sessions in the first year following surgery, with subsequent yearly follow-up appointments up to 5 years after the procedure.

This study was approved by the Medical Ethical committee (METC Zuidwest Holland 19–005). Data was collected up to the 27th of December 2022.

### Standard Psychosocial-Behavioral Evaluation

Before being accepted for treatment, patients were screened according to the International Federation for the Surgery of Obesity criteria by a multidisciplinary team comprising of a medical doctor, dietitian, and a psychologist [1]. In this study, patients were evaluated by one of the seven psychologists working at the clinic in The Hague.

The psychosocial-behavioral assessment protocol implemented at the clinic starts with the completion of two questionnaires by the patient, prior to undergoing the preoperative screening process. The first questionnaire is the Brief Symptom Inventory, which encompasses nine symptom dimensions of depression, namely somatization, obsession-compulsion, interpersonal sensitivity, depression, anxiety, hostility, phobic anxiety, paranoid ideation, and psychoticism [9]. The second questionnaire is an intake questionnaire specifically developed by the clinic, designed to gather information on various subjects including the patient’s eating pattern, symptoms of binge eating disorder or bulimia, previous psychological illnesses, and/or treatment, and the use of alcohol, drugs, or tobacco. The psychologist then conducts a semi-structured interview, which covers topics such as the reasons for seeking MBS, the patient’s expectations for the treatment, and any potential pitfalls, such as emotional-eating or irregular work patterns. The interview also evaluates the patient’s social support system, mood and mood disorders, the presence of social phobia, post-traumatic stress disorder, psychosis, (binge) eating disorders or other chronic psychiatric disorders, substance use, motivation for behavioral change, and the patient’s suitability for group therapy. Additionally, the psychosocial-behavioral evaluation aims to identify any potential postoperative risk factors. Finally, the patient’s case is discussed by the multidisciplinary team, who make a recommendation for treatment (“positive,” “postponed decision,” or “negative”). Alongside this recommendation, tailored propositions for subsequent treatment are provided, considering the specific pitfalls and areas for improvement identified in each patient. For example, proposing a preliminary consultation to address emotional eating tendencies—a potential pitfall that necessitates attention but does not warrant a negative screening recommendation. Reasons for a “postponed decision” include the patient’s requirement for guidance from a registered dietitian, as well as the need for psychological evaluation and treatment, for conditions such as depressive disorder or (symptoms of) binge eating disorder, prior to the patient being suitable for surgery.

### Cleveland Clinic Behavioral Rating System

The CCBRS evaluates nine domains that are believed to be important for the patient’s psychosocial consultation, including consent, expectations, social support, adherence, coping/stressors, mental health, substance use/abuse/dependence, eating behaviors, and overall impression [4]. Each domain is assessed by a licensed psychologist using a five-point Likert scale. The ratings were classified as follows: 5 for “excellent,” indicating no concerns and no further psychological follow-up is needed unless problems arise in the future; 4 for “good,” indicating a manageable problem with concerns that can be addressed without significant intervention; 3 for “fair,” indicating the presence of concerns or risk factors that are reasonably controlled or managed, with a balance between the patient’s strengths and areas for improvement; 2 for “guarded,” indicating the strong recommendation for intervention before proceeding and requiring discussion in multidisciplinary rounds; and 1 for “poor,” indicating an inappropriate risk that very likely outweighs benefits.

In this study, all psychologists were instructed to administer the CCBRS to each patient in addition to the standard psychosocial-behavioral evaluation that was conducted during the screening process. Prior to the initiation of the study, the psychologists underwent a training process, which involved written instructions detailing how to accurately complete the CCBRS assessment. Furthermore, a collaborative session involving all psychologists was conducted to review and discuss examples, thereby reinforcing the standardized approach. Additionally, a subsequent session was organized to verify consistent implementation of the assessment methodology across the team. These efforts were aimed at maintaining uniformity in the application of the CCBRS and enhancing its reliability. Lastly, it should be noted that the results obtained from the CCBRS had no impact on the ultimate decision regarding a patient’s suitability for surgery in this study.

### Body Weight and Other Parameters

The study data were collected from the prospectively maintained database of the clinic. It encompassed patient demographic information (age and gender), along with the presence of obesity-associated medical problems such as hypertension, diabetes mellitus, hyperlipidemia, and obstructive sleep apnea. Details regarding the type of surgery were also recorded, and whether it was the primary or secondary bariatric-metabolic procedure (surgical revision). Additionally, measurements of height and weight were taken during the initial screening at the clinic and weight was also measured at the start of the preoperative care program, and during subsequent follow-up appointments up to 5 years after surgery (at intervals of 3, 6, 12, 18, 24, 36, 48, and 60 months). From March 2020 to August 2021, patients provided self-reported bodyweight due to telephone appointments prompted by the COVID-19 pandemic. Baseline and all follow-up measurements were used to calculate (change in) body mass index (BMI) and percentage total weight loss (%TWL): %TWL = ((preoperative weight – weight at follow-up)/preoperative weight) × 100%.

### Statistical Analysis

Descriptive statistics were used to summarize the baseline characteristics of the population and CCBRS scores. Different groups were compared: patients scored with the CCBRS versus those who were not, patients who underwent MBS versus those who did not, and patients who attended versus those who did not attend the 5-year follow-up appointment. Continuous variables were analyzed using Student’s *t* test and binary variables with the *χ*^2^ test.

Visual representations of the %TWL over time were generated for the entire study population, as well as for CCBRS scores for the “overall impression” domain, where a higher %TWL corresponds to more weight loss. Linear mixed models were used to analyze %TWL over time up to 5 years after MBS. Subsequently, a linear mixed model was conducted to assess the association between %TWL and each separate domain of the CCBRS. Five study groups were established based on the CCBRS score categories assigned by the psychologist (5: “excellent,” 4: “good,” 3: “fair,” 2: “guarded,” and 1: “poor”). First, to evaluate differences in %TWL between the five study groups, a crude linear mixed model was conducted with CCBRS score categories as predictor and %TWL as outcome. Second, an adjusted model was conducted with age at day of surgery, gender, type of surgery, surgical revision, and preoperative BMI as confounders.

For each patient, the number of attended follow-up appointments from years 1 to 5 was calculated. Individuals who attended all follow-up visits were assigned a score of five, while those who missed all sessions received a score of zero. This resulted in the creation of six distinct groups based on attendance score. Ordinal regression analyses were used to examine the association between CCBRS scores and the attendance scores up to the 5-year follow-up visit. Again, a crude model was initially created, which was then adjusted for age at day of surgery, gender, type of surgery, surgical revision, and preoperative BMI. The outcome category “excellent” was used as the reference category for both the linear mixed models and ordinal regression analyses. The log odds and corresponding confidence intervals of each model were converted to odds ratios (OR) and presented in the tables.

Above analyses were conducted using IBM SPSS Statistics for Mac, version 27.0. A *p*-value of less than 0.05 was considered statistically significant.

## Results

### Study Population

**Fig. 1:**
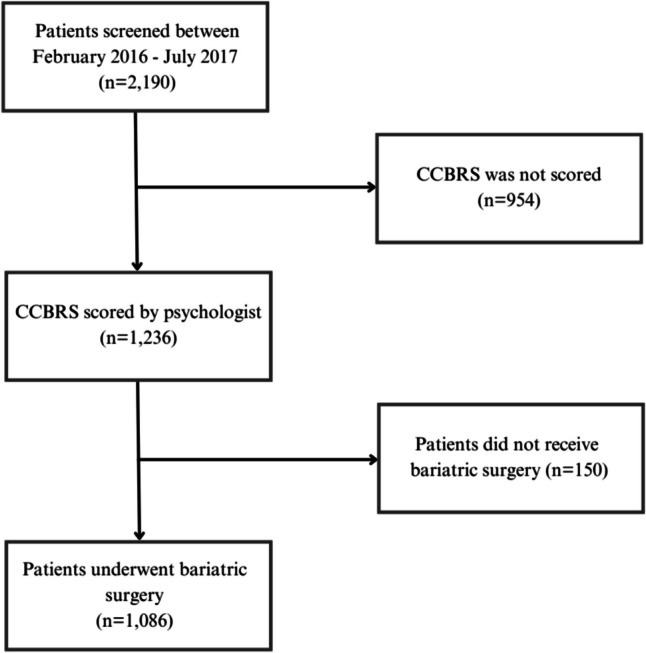
Flowchart of the study population. CCBRS: Cleveland Clinic Behavioral Rating System

A total of 2190 patients were screened during a period extending from February 2016 to July 2017. Of these, 954 patients (44%) were excluded from the study as their CCBRS scores were not recorded (Fig. 1). The baseline characteristics of patients scored with the CCBRS and those not scored were comparable for both groups (Table 1). In 1236 patients a CCBRS score was available, and these patients were included in the study. The mean age of the study population was 43.2 (± 11.7) years, with 75.6% of the participants being female (Table 2). The mean BMI at screening was 43.5 (± 5.7) kg/m^2^.

**Table 1:** Baseline characteristics, comparing patients who were and were not scored with the CCBRS. Presented as mean ± standard deviation or *n* (%)

	All (*n* = 2190)	Scored with CCBRS (*n* = 1236)	Not scored with CCBRS (*n* = 954)	*P***-**value
Age at screening, years	43.6 ± 11.8	43.3 ± 11.7	44.0 ± 12.0	.155
Sex				
Female	1657 (75.7)	935 (75.6)	722 (75.7)	.985
Weight, kg				
Preoperative screening	123.6 ± 21.4	124.1 ± 21.2	122.9 ± 21.6	.207
Body mass index, kg/m^2^				
Preoperative screening	43.3 ± 5.7	43.5 ± 5.7	43.1 ± 5.7	.181
Associated medical problems				
Hypertension	761 (38.0)	445 (38.3)	316 (37.6)	.743
Type II diabetes	429 (21.4)	253 (21.8)	176 (20.9)	.649
Dyslipidemia	429 (21.4)	239 (20.6)	190 (22.6)	.276
Sleep apnea	365 (18.2)	200 (17.2)	165 (19.6)	.168
Osteoarthritis	301 (15.0)	183 (15.7)	118 (14.0)	.288

*CCBRS* Cleveland Clinic Behavioral Rating System

**Table 2:** Baseline characteristics of the included population, comparing patients who had surgery with patients who did not have surgery. Presented as mean ± standard deviation or *n* (%)

	All (*n* = 1236)	Surgery (*n* = 1086)	No surgery (*n* = 150)	*P***-**value
Age at screening, years	43.2 ± 11.7	43.2 ± 11.6	43.7 ± 12.5	.708
Sex				<
Female	935 (75.6)	840 (77.3)	95 (63.3)	.001
Weight, kg				
Preoperative screening	124.1 ± 21.2	124.1 ± 20.7	124.0 ± 25.7	.926
Body mass index, kg/m^2^				
Preoperative screening	43.5 ± 5.7	43.5 ± 5.6	43.0 ± 6.8	.360
Start preoperative counseling program		43.7 ± 5.6		
Associated medical problems				
Hypertension	445 (38.3)	398 (37.6)	47 (45.2)	.129
Type II diabetes	253 (21.8)	234 (22.1)	19 (18.3)	.364
Dyslipidemia	239 (20.6)	216 (20.4)	23 (22.1)	.682
Sleep apnea	200 (17.2)	178 (16.8)	22 (21.2)	.264
Osteoarthritis	183 (15.7)	171 (16.2)	12 (11.5)	.217
Surgical method				
Roux-en-Y gastric bypass		769 (70.8)		
Sleeve gastrectomy		294 (27.1)		
Single anastomosis duodenal ileal bypass		14 (1.3)		
One-anastomosis gastric bypass		6 (0.6)		
Elongated Roux-en-Y gastric bypass		3 (0.3)		
Primary procedure		1038 (95.6)		
Secondary procedure		48 (4.4)		

A total of 1086 patients (88%) were assessed with the CCBRS and underwent subsequent MBS, while 150 patients (12%) did not undergo surgery. RYGB was the most frequently performed surgical procedure (*n* = 769, 70.8%). The group of patients who underwent surgery had a higher percentage of female patients compared to the group who did not undergo surgery (77.3% versus 63.3%, Table 2).

### Distribution of CCBRS Scores

Upon examination of the assigned scores for each CCBRS domain, the score of “poor” was assigned the least frequently, with a range of one to nineteen patients per domain (Table 3). Most patients received a score of “good” on seven out of the nine domains. Only in the “consent” and “chemical/alcohol abuse/dependence” domains did most patients receive a score of “excellent.” Patients who underwent MBS were assigned higher scores in every domain, when compared to those who did not receive surgery.

**Table 3:** The assigned scores of all included patients for each CCBRS domain. Presented as number (%)

CCBRS domain	Population	CCBRS score				
		Poor	Guarded	Fair	Good	Excellent
1. Consent (includes capacity to consent, possible cognitive impairment, understanding of risks, benefits, alternative treatment)	Total	11 (0.9)	59 (4.8)	247 (20.0)	459 (37.1)	460 (37.2)
	Surgery	9 (0.8)	46 (4.2)	203 (18.7)	409 (37.7)	419 (38.6)
	No surgery	2 (1.3)	13 (8.7)	44 (29.3)	50 (33.3)	41 (27.3)
2. Expectations (includes realistic nature of surgery, recovery, early transition, weight loss goals, effect on relationships, quality of life, long-term outcome)	Total	1 (0.1)	44 (3.6)	285 (23.1)	632 (51.1)	274 (22.2)
	Surgery	0	30 (2.8)	231 (21.3)	573 (52.8)	252 (23.2)
	No surgery	1 (0.7)	14 (9.3)	54 (36.0)	59 (39.3)	22 (14.7)
3. Social support (includes spouse or significant other, children, family members, friends, employer, co-workers; also includes past conversations with bariatric patients, attendance at support groups)	Total	5 (0.4)	70 (5.7)	228 (18.4)	610 (49.4)	323 (26.1)
	Surgery	3 (0.3)	52 (4.8)	188 (17.3)	547 (50.4)	296 (27.3)
	No surgery	2 (1.3)	18 (12.0)	40 (26.7)	63 (42.0)	27 (18.0)
4. Mental health (includes psychiatric diagnosis and severity and duration of diagnosis; determination should be based on effect of illness on cognitive capacity, present stability/instability of illness, current treatment, adherence to treatment recommendations, psychosocial stresses that could affect illness and patient insight)	Total	14 (1.1)	101 (8.2)	318 (25.7)	494 (40.0)	308 (24.9)
	Surgery	8 (0.7)	80 (7.4)	272 (25.0)	445 (41.0)	280 (25.8)
	No surgery	6 (4.0)	21 (14.0)	46 (30.7)	49 (32.7)	28 (18.7)
5. Chemical/alcohol abuse/dependence (includes use, abuse, and dependence on alcohol, prescription drugs, and illicit drugs; include history and present use in determination; if history positive, consider period of sobriety and relapse risk; weigh tobacco use/likelihood of quitting in assessment)	Total	4 (0.3)	37 (3.0)	197 (15.9)	415 (33.6)	583 (47.2)
	Surgery	3 (0.3)	29 (2.7)	164 (15.1)	363 (33.4)	527 (48.5)
	No surgery	1 (0.7)	8 (5.3)	33 (22.0)	52 (34.7)	56 (37.3)
6. Eating behaviors (includes binge eating behaviors, night eating behaviors, compensatory behaviors, history of eating disordered behaviors, and problematic outcomes from past dieting attempts; consider behaviors [e.g., “grazing,” high-calorie beverage consumption] that might affect outcome)	Total	19 (1.5)	135 (10.9)	498 (40.3)	545 (44.1)	39 (3.2)
	Surgery	12 (1.1)	109 (10.0)	429 (39.5)	501 (46.1)	35 (3.2)
	No surgery	7 (4.7)	26 (17.3)	69 (46.0)	44 (29.3)	4 (2.7)
7. Adherence (includes adherence during previous dieting attempts, adherence with past psychological/psychiatric interventions, adherence with medical recommendations, and likely adherence with tobacco prohibition and program protocol)	Total	13 (1.1)	79 (6.4)	414 (33.5)	587 (47.5)	142 (11.5)
	Surgery	11 (1.0)	59 (5.4)	351 (32.3)	531 (48.9)	133 (12.2)
	No surgery	2 (1.3)	20 (13.3)	63 (42.0)	56 (37.3)	9 (6.0)
8. Coping/stressors (includes an assessment of coping resources in the context of situational stressors)	Total	11 (0.9)	115 (9.3)	412 (33.3)	593 (48.0)	104 (8.4)
	Surgery	7 (0.6)	85 (7.8)	356 (32.8)	537 (49.4)	100 (9.2)
	No surgery	4 (2.7)	30 (20.0)	56 (37.3)	56 (37.3)	4 (2.7)
9. Overall impression	Total	12 (1.0)	110 (8.9)	365 (29.5)	596 (48.2)	150 (12.1)
	Surgery	7 (0.6)	76 (7.0)	307 (28.3)	548 (50.5)	145 (13.4)
	No surgery	5 (3.3)	34 (22.7)	58 (38.7)	48 (32.0)	5 (3.3)

Total population *n* = 1236, surgery *n* = 1086, no surgery *n* = 150

### Postoperative Weight Loss

**Fig. 2:**
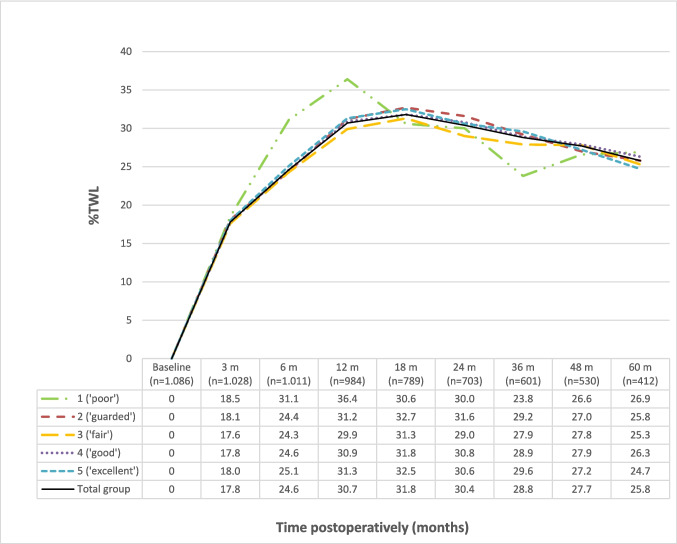
Percentage total weight loss (%TWL) for the CCBRS-score categories in the domain “overall impression.” m, months after surgery; n, number of patients

Highest weight loss was attained eighteen months after surgery, with an average %TWL of 31.8 (± 8.7, Fig. 2) and mean change in BMI of 13.9 kg/m^2^ (± 4.3). Five years after surgery this percentage had decreased to a mean %TWL of 25.8 (± 9.9) and mean change in BMI of 11.0 kg/m^2^ (± 4.6).

### Association Between Weight Loss and CCBRS Scores

The weight loss for each CCBRS score within the “overall impression” domain is graphically presented in Fig. 2. The CCBRS score category with the highest %TWL varied depending on the follow-up time point. The group categorized as “poor” in this domain consisted of four patients at the 6-month follow-up and two patients at the 5-year follow-up.

Crude and adjusted linear mixed models were performed for each domain of the CCBRS (Table 4). Adjusted linear mixed model analysis showed that patients with a “fair” score had a lower %TWL over time compared to the reference group “excellent” in the domains “consent” (*β* − 1.30; 95% CI [− 2.41 to − 0.19]) and “mental health” (*β* − 1.30; 95% CI [− 2.41 to − 0.19]). However, for none of these domains, there were significant or clinically relevant differences with the other groups. For example, within the “consent” domain, patients with a “good” score did not exhibit greater weight loss compared to patients scoring “poor” or “guarded.” In the domain “social support,” the “good” group had a lower %TWL over time compared to the reference group (*β* − 1.12 [− 2.05 to − 0.19]). No statistically significant difference in %TWL over time was observed for any other groups and domains.

**Table 4:** Percentage total weight loss over time using linear mixed models for each CCBRS domain, with group “excellent” used as the reference category for each model, presented as *β*-coefficient [95% confidence interval]

CCBRS domain and groups	Crude model	Adjusted model^†^
1. Consent	27.23 [26.56–27.90]	19.47 [15.15–23.79]
• Poor	0.96 [− 3.99–5.91]	0.00 [− 4.66–4.66]
• Guarded	− 0.15 [− 2.33–2.02]	− 0.20 [− 2.25–1.84]
• Fair	− 1.52 [− 2.70 to − 0.34]*	− 1.30 [− 2.41 to − 0.19]*
• Good	− 0.66 [− 1.61–0.30]	− 0.77 [− 1.66–0.13]
2. Expectations	26.55 [25.68–27.42]	18.94 [14.59–23.29]
• Poor	–	–
• Guarded	− 1.48 − 4.29–1.34]	− 1.65 [− 4.30–1.01]
• Fair	0.13 [− 1.13–1.39]	0.09 [− 1.10–1.27]
• Good	0.31 [− 0.73–1.35]	0.09 [− 0.89–1.07]
3. Social support	27.62 [26.83–28.42]	19.58 [15.24 –23.91]
• Poor	5.09 [-4.39–14.56]	4.82 [− 4.05–13.70]
• Guarded	− 1.58 [− 3.69–0.53]	− 1.41 [− 3.39–0.57]
• Fair	− 1.20 [− 2.49–0.96]	− 0.91 [− 2.13 –0.30]
• Good	− 1.29 [− 2.27 to − 0.30]*	− 1.12 [− 2.05 to − 0.19]*
4. Mental health	27.23 [26.41–28.04]	19.64 [15.27–24.01]
• Poor	1.78 [− 3.88–7.43]	2.14 [− 3.16–7.45]
• Guarded	− 0.37 [− 2.15–1.40]	− 0.69 [− 2.36–0.97]
• Fair	− 1.10 [− 2.27–0.07]	− 1.30 [− 2.41 to − 0.19]*
• Good	− 0.55 [− 1.59–0.49]	− 0.74 [− 1.73–0.24]
5. Chemical/alcohol abuse/dependence	26.47 [25.87–27.07]	18.69 [14.39–23.00]
• Poor	− 6.72 [− 14.69–1.26]	− 3.89 [− 11.41–3.64]
• Guarded	1.48 [− 1.15–4.10]	1.34 [− 1.15–3.84]
• Fair	0.37 [− 0.89–1.62]	0.62 [− 0.56–1.81]
• Good	0.48 [− 0.46–1.41]	0.43 [− 0.45–1.31]
6. Eating behaviors	27.49 [25.21–29.78]	19.75 [15.05–24.45]
• Poor	0.61 [− 4.08–5.30]	1.17 [− 3.23–5.58]
• Guarded	0.11 [− 2.54–2.77]	− 0.30 [− 2.81–2.20]
• Fair	− 1.03 [− 3.41–1.36]	− 1.11 [− 3.35–1.14]
• Good	− 0.86 [− 3.23–1.51]	− 1.01 [− 3.24–1.21]
7. Adherence	26.99 [25.80–28.18]	19.09 [14.67–23.51]
• Poor	− 4.45 [− 9.17–0.27]	− 2.12 [− 6.59–2.34]
• Guarded	0.57 [− 1.67–2.80]	0.23 [− 1.87–2.34]
• Fair	− 0.86 [− 2.26–0.54]	− 0.77 [− 2.10–0.55]
• Good	0.03 [− 1.30–1.35]	0.03 [− 1.22–1.28]
8. Coping/stressors	27.18 [25.82–28.55]	19.52 [15.10–23.94]
• Poor	0.31 [− 5.96–6.58]	0.43 [− 5.46–6.31]
• Guarded	− 0.72 [− 2.76–1.33]	− 1.15 [− 3.08–0.78]
• Fair	− 0.92 [− 2.47–0.64]	− 1.23 [− 2.70–0.23]
• Good	− 0.28 [− 1.77–1.21]	− 0.63 [− 2.03–0.77]
9. Overall impression	27.23 [26.09–28.36]	19.34 [14.95–23.72]
• Poor	3.26 [− 2.97–9.50]	4.11 [− 1.74–9.96]
• Guarded	− 0.49 [− 2.45–1.47]	− 0.69 [− 2.54–1.15]
• Fair	− 1.05 [− 2.44–0.34]	− 0.92 [− 2.23–0.38]
• Good	− 0.41 [− 1.69–0.86]	− 0.71 [− 1.91–0.49]

*CCBRS*, Cleveland Clinic Behavioral Rating System

^**†**^Linear mixed model corrected for age at day of surgery, gender, type of surgery, redo surgery, and preoperative BMI. * *p* < 0.05

### Association Between Attendance to Follow-up and CCBRS Scores

The attendance at the 5-year follow-up after surgery was 37.9% (Table 5). Table 6 provides an overview of the number of yearly follow-up appointments attended per patient. Notably, 6.4% of patients attended none of the appointments during these five years, while 24.1% attended all five sessions. Table 7 presents the baseline characteristics of patients who attended and did not attend the 5-year follow-up appointment. Those lost to follow-up were younger (42.8 years versus to 45.6 years on average) and had higher preoperative weight (126.1 kg versus to 121.1 kg) and BMI (44.1 kg/m^2^ versus to 42.7 kg/m^2^) compared to patients who attended the 5-year follow-up. Crude and adjusted ordinal regression analyses were performed for each CCBRS domain (Table 8). The analyses illustrated that patients who scored “poor” in the domains “consent,” “coping/stressors,” and “overall impression” were less likely to attend the postoperative yearly sessions when compared to the reference group that scored “excellent” in these domains (OR 0.15 [0.04–0.57], 0.19 [0.05–0.77] and 0.15 [0.04–0.60] respectively). In the “adherence” domain, both the “poor” and “guarded” group were less likely to attend the follow-up appointments (OR 0.25 [0.08–0.76] and 0.48 [0.27–0.84]) and for the “expectations” domain this was only the “guarded” group (OR 0.48 [0.24–0.99]). No statistically significant differences in attendance were observed for the other CCBRS scores or domains.

**Table 5:** Compliance to postoperative follow-up appointments, presented as number (%)

	Baseline	3 months	6 months	12 months	18 months	24 months	36 months	48 months	60 months
Present at follow-up	1.086	1.028	1.011	984	789	703	601	530	412
	(100)	(94.7)	(93.1)	(90.6)	(72.7)	(64.7)	(55.3)	(48.8)	(37.9)

**Table 6:** Compliance to the yearly postoperative follow-up appointments, presented as number (%)

Number of appointments attended per patient	
0	67 (6.4)
1	170 (16.2)
2	160 (15.3)
3	166 (15.8)
4	233 (22.2)
5	253 (24.1)

**Table 7:** Baseline characteristics of patients who did and did not attend the five-year follow-up appointment. Presented as mean ± standard deviation or *n* (%)

	Attended (*n* = 412)	Not attended (*n* = 674)	*P*-value
Age at surgery, years	45.6 ± 10.5	42.8 ± 12.1	< .001
Sex			
Female	324 (78.6)	516 (76.6)	.426
Weight, kg			
Preoperative screening	121.1 ± 18.9	126.1 ± 21.6	< .001
Body mass index, kg/m^2^			
Preoperative screening	42.7 ± 5.1	44.1 ± 5.8	< .001
Associated medical problems			
Hypertension	169 (41.0)	229 (35.4)	.068
Type II diabetes	95 (23.1)	139 (21.5)	.556
Dyslipidemia	82 (19.9)	134 (20.7)	.741
Sleep apnea	73 (17.7)	105 (16.3)	.535
Osteoarthritis	74 (18.0)	97 (15.0)	.204

*CCBRS* Cleveland Clinic Behavioral Rating System

**Table 8:** Odds ratio for compliance to follow-up up to 5 years after surgery using ordinal regression analysis for each CCBRS domain, with group “excellent” used as the reference category for each model, presented as OR [95% confidence interval]

CCBRS domain and groups	Crude regression	Adjusted regression^†^
1. Consent		
• Poor	0.23 [0.07–0.76]*	0.15 [0.04–0.57]*
• Guarded	0.75 [0.44–1.28]	0.87 [0.50–1.52]
• Fair	0.80 [0.59–1.07]	0.84 [0.62–1.13]
• Good	0.83 [0.65–1.06]	0.81 [0.63–1.04]
2. Expectations		
• Poor	-	-
• Guarded	0.45 [0.23–0.88]*	0.48 [0.24–0.99]*
• Fair	1.01 [0.74–1.38]	0.95 [0.69–1.31]
• Good	1.08 [0.83–1.40]	0.94 [0.72–1.23]
3. Social support		
• Poor	0.66 [0.09–4.90]	1.02 [0.11–9.61]
• Guarded	0.78 [0.46–1.31]	0.89 [0.52–1.52]
• Fair	0.73 [0.53–1.01]	0.80 [0.58–1.12]
• Good	0.97 [0.76–1.24]	0.97 [0.75–1.25]
4. Mental health		
• Poor	0.27 [0.08–0.93]*	0.36 [0.10–1.34]
• Guarded	1.18 [0.55–1.31]	0.85 [0.54–1.33]
• Fair	0.86 [0.64–1.16]	0.90 [0.66–1.22]
• Good	1.01 [0.77–1.31]	0.99 [0.76–1.30]
5. Chemical/alcohol abuse/dependence		
• Poor	0.42 [0.06–3.08]	0.24 [0.03–1.82]
• Guarded	0.66 [0.34–1.27]	0.59 [0.30–1.15]
• Fair	0.78 [0.57–1.07]	0.77 [0.56–1.06]
• Good	1.20 [0.94–1.52]	1.18 [0.93–1.51]
6. Eating behaviors		
• Poor	0.53 [0.17–1.68]	0.56 [0.17–1.81]
• Guarded	0.63 [0.32–1.23]	0.92 [0.46–1.84]
• Fair	0.90 [0.49–1.65]	1.19 [0.64–2.22]
• Good	1.06 [0.58–1.94]	1.26 [0.68–2.34]
**7. Adherence**		
• Poor	0.22 [0.07–0.67]*	0.25 [0.08–0.76]*
• Guarded	0.40 [0.23–0.68]*	0.48 [0.27–0.84]*
• Fair	0.73 [0.51–1.03]	0.84 [0.58–1.20]
• Good	0.84 [0.60–1.18]	0.89 [0.63–1.26]
8. Coping/stressors		
• Poor	0.27 [0.07–1.05]	0.19 [0.05–0.77]*
• Guarded	0.87 [0.52–1.44]	0.96 [0.57–1.63]
• Fair	0.98 [0.66–1.44]	1.05 [0.70–1.57]
• Good	1.18 [0.81–1.72]	1.22 [0.83–1.81]
9. Overall impression		
• Poor	0.18 [0.05–0.71]*	0.15 [0.04–0.60]*
• Guarded	0.66 [0.40–1.07]	0.70 [0.42–1.15]
• Fair	0.85 [0.60–1.20]	0.96 [0.67–1.37]
• Good	0.99 [0.72–1.37]	1.04 [0.75–1.45]

*CCBRS* Cleveland Clinic Behavioral Rating System

^**†**^Ordinal regression analysis corrected for age at day of surgery, gender, type of surgery, redo surgery and preoperative BMI. *p < 0.05

## Discussion

The aim of the present study was to evaluate the predictive value of the CCBRS on weight loss and follow-up attendance up to 5 years after MBS. Postoperative weight loss and attendance were compared for different CCBRS scores in each domain separately. The findings revealed that the CCBRS did not predict weight loss, nor follow-up attendance up to 5 years after surgery in this study, which supports the continued need for a comprehensive and personalized approach.

Most patients in this study were rated as having a “good” score across most of the domains assessed by the CCBRS, while only a small number of patients were classified as “poor” in all domains. These findings are consistent with prior research, which reported that most patients received scores of “fair” or “good” across various domains, according to the CCBRS developers [4]. However, the proportion of patients receiving an “excellent” rating in the present study was significantly higher than in previous research, ranging from 3.2 to 47.2%, depending on the domain assessed. This difference may be attributed to variations in the study population from different geographic regions, where cultural and social determinants may impact patients’ health status and functional outcomes. Moreover, it should be noted that the scoring of the CCBRS is subjective, and the observed differences could also be attributed to inter-rater variations between different regions.

The findings of the present study indicate that the predictive value of the CCBRS for weight loss up to 5 years after surgery was limited. In three out of nine domains, only one group demonstrated a statistically significant difference in postoperative weight loss when compared to the reference group. The observed differences in %TWL are considered not clinically significant, as they amount to only a small difference ranging from 1.21 to 1.30%. Considering the average preoperative weight of 124 kg, this difference in weight loss translates to a mere 1.5–1.6 kg, which will not to make a meaningful impact on a patient’s weight or health outcomes. Furthermore, the beta coefficients observed in all nine domains did not consistently favor one of the CCBRS scores, which suggests that there is no clear association between the CCBRS score and postoperative weight loss. These findings are consistent with prior studies, reaffirming the ongoing limited prognostic value of preoperative psychological conditions in general in relation to weight loss after MBS [10, 11].

Only 24.1% of patients attended all five yearly follow-up appointments and 37.9% attended the 5-year follow-up appointment. It is known that attendance to follow-up appointments after MBS is a challenge in most bariatric programs and research has consistently shown that attrition rates vary significantly across studies [12]. In addition, the 2018 Fourth IFSO Global Registry Report revealed that even at 1-year follow-up, data for weight loss were available for less than half of the patients and that analyses relying on follow-up data should be interpreted bearing this consideration in mind [13]. To limit the impact of loss to follow-up, all available follow-up time points were included in the analysis. Linear mixed models and ordinal regression analyses were used to comprehensively analyze the data, instead of focusing on the outcomes solely 5 years after surgery. Moreover, baseline characteristics of patients who were lost to follow-up were largely comparable to those who attended the 5-year appointment, except for differences in age and preoperative weight/BMI. This similarity suggests comparability between the groups, suggesting that the sample is representative and less susceptible to bias. Previous research also suggests that younger individuals are more likely to be lost to follow-up [14]. Nevertheless, the significant loss to follow-up must be considered when interpreting the results.

Previous research found that the CCBRS domains “social support,” “coping,” and “overall impression” were associated with attendance to follow-up 2 years after MBS [5], while in a more recent study the domain “social support” was not associated with attendance up to 2 years after surgery [6]. The current study suggests that there is no clear association between CCBRS scores and attendance to follow-up. Few significant associations were found, though they were never observed across all groups within a single domain. These significant associations were predominantly in the “poor” group, which is characterized by a small sample size in this cohort. The percentage of patients categorized as “poor” ranged from 0.1 to 1.5% across the different domains, which is similar to the proportion reported in prior research, where 2.6% of patients were classified as “poor” [4]. However, this low proportion may have negatively skewed the data and poses a challenge for making definitive statements regarding weight loss and attendance to follow-up, especially in this subgroup, as it increases the susceptibility of the results to individual variations in outcomes. Therefore, caution should be exercised when interpreting the results of this group. It is also important to note that the groups showing significant differences in attendance were different from the groups that showed differences in weight loss. The statistically significant differences observed in certain groups for weight loss and attendance could potentially be attributed to chance. The likelihood of accidentally identifying a significant difference increases as more analyses are conducted on a larger number of groups, even if such differences may not be clinically relevant. It is probable that if the analyses were repeated on a different cohort, other groups and domains would be found to be statistically different by chance alone.

The association between preoperative psychological factors and postoperative outcomes in MBS remains complicated. According to international guidelines, patients with severe psychological concerns should undergo thorough mental health evaluations and/or treatment before MBS [1]. Therefore, the absence of an association between CCBRS domains and outcomes after MBS could also suggest the success of preoperative treatment, making it difficult to establish an effect of the initial diagnosis on postoperative outcomes. Future research could consider conducting a follow-up assessment after preoperative treatment among patients with psychological concerns at initial screening. This reassessment could provide a more comprehensive understanding of the association between preoperative psychological conditions and postoperative outcomes.

Furthermore, it is important to note that the CCBRS specifically focuses on assessing psychological factors, while obesity is recognized as a chronic and multifaceted condition influenced by a wide array of other factors, including eating behaviors, physical activity, genetic risk, poor sleep patterns, health conditions, medication usage, and the individuals’ environment [15]. This complex interplay contributes to the development of obesity and consequently impacts weight loss following BMS. It is conceivable that the interaction among these factors makes the identification of isolated predictors of weight loss after BMS challenging.

Therefore, we recommend that future research should include other outcomes, as weight loss and attendance might not always represent the most critical aspects. For example, a previous study demonstrated the predictive value of the CCBRS on postoperative quality of life, depression, and anxiety [5]. Furthermore, alongside the CCBRS, other standardized preoperative assessment tools, such as the Bariatric Interprofessional Psychosocial Assessment of Suitability Scale (BIPASS) scale [16], have found to be predictive in assessing post-surgical binge eating symptomatology and mental health-related quality of life.

Despite the limitations in predicting weight loss and attendance in this study, the CCBRS remains valuable for conducting a systematic psychological evaluation of individuals seeking MBS and covers most of the recommendations outlined in the presurgical psychosocial evaluation guideline by Sogg et al. [2]. The CCBRS aids in identifying patients’ strengths and areas for improvement, thereby facilitating enhanced interdisciplinary communication. Moreover, the CCBRS supports the ongoing implementation of a personalized approach to patient care.

One of the strengths of this study is the large sample size of 1236 patients and a relatively long-term follow-up, in contrast to previous studies with only 179 to 415 included patients and 2-year follow-up [4–6]. Only one prior study has investigated the association between each CCBRS domain and weight loss and attendance [5], whereas the other two previous studies only examined the domains of “social support” and “overall impression” in relation to weight loss and attendance [4, 6]. The analysis of each of the CCBRS domains separately allows for a more nuanced understanding of the relationship between weight loss and various aspects of patients’ functioning, rather than treating patients’ overall functioning as a single construct. For example, a patient may have good overall functioning but struggle with emotional eating, which could have a significant impact on their ability to lose weight. An additional limitation is that participants from only one clinic in The Netherlands were included, which limits the generalizability of the findings to other populations. The results may not be representative of experiences and behaviors in other cultures or regions, and the results might not align with previous research findings from different populations or settings. Another study limitation relates to the self-reported bodyweight measurements necessitated by the COVID-19 pandemic. While acknowledging the potential for data bias, it is important to highlight that a recently published study, originating from the same clinic as well, uncovered a marginal mean difference of 0.75–1.17 kg between self-reported and clinic-measured weights [17]. This finding underscores the patients’ accuracy in reporting their weight, which contributes to minimizing potential biases. An additional limitation is the lack of data on other variables that could influence weight loss such as postoperative short- and long-term complications. A final limitation of this study is the exclusion of 44% of study participants due to an inadvertent oversight by psychologists, who unintentionally did not administer the CCBRS after initial screening for almost half of the patients. This situation occurred randomly and without intentional bias, which is also reflected in the comparable baseline characteristics between these two groups and therefore decreases the chances of selection bias.

## Conclusion

The CCBRS did not predict weight loss or follow-up attendance up to 5 years after MBS in this cohort. It is important to acknowledge the study’s limitations, such as considerable loss to follow-up. A comprehensive and personalized approach that incorporates various patient traits and factors is necessary to achieve optimal preoperative selection of patients and desirable outcomes after surgery. The CCBRS remains valuable for the preoperative psychological evaluation of individuals undergoing MBS. It promotes interdisciplinary communication by identifying the patients’ strengths and areas for improvement, which aligns with its intended purpose.

## Abbreviations

BMI: Body mass index
CCBRS: Cleveland Clinic Behavioral Rating System
MBS: Metabolic and bariatric surgery
OR: Odds ratio
RYGB: Roux-en-Y gastric bypass
TWL: Total weight loss
